# Improving the Performance of SVM-RFE to Select Genes in Microarray Data

**DOI:** 10.1186/1471-2105-7-S2-S12

**Published:** 2006-09-26

**Authors:** Yuanyuan Ding, Dawn Wilkins

**Affiliations:** 1 Computer & Information Science Department, The University of Mississippi, University, MS, USA

## Abstract

**Background:**

Recursive Feature Elimination is a common and well-studied method for reducing the number of attributes used for further analysis or development of prediction models. The effectiveness of the RFE algorithm is generally considered excellent, but the primary obstacle in using it is the amount of computational power required.

**Results:**

Here we introduce a variant of RFE which employs ideas from simulated annealing. The goal of the algorithm is to improve the computational performance of recursive feature elimination by eliminating chunks of features at a time with as little effect on the quality of the reduced feature set as possible. The algorithm has been tested on several large gene expression data sets. The RFE algorithm is implemented using a Support Vector Machine to assist in identifying the least useful gene(s) to eliminate.

**Conclusion:**

The algorithm is simple and efficient and generates a set of attributes that is very similar to the set produced by RFE.

## Background

In many machine learning applications, a prediction is to be made from a data set of historical information. Gene expression data sets have been constructed with the goal of predicting whether or not disease is present (e.g. colon cancer), or which type of disease exists in the patient. One of the primary difficulties in working with gene expression data sets is the large number of attributes (genes). A major focus of gene expression analysis is in the area of feature selection or dimension reduction. Most of the algorithms for elucidating models for prediction are less effective when the number of genes is too large. There are many approaches for reducing the size of the feature set, and among them is recursive feature elimination (RFE). The idea of RFE is to start with all features, select the "least useful" feature (using some metric or heuristic), remove that feature, and repeat until some stopping condition is met. There are many variations of RFE based on how the feature to be removed is selected, and when to stop.

RFE is well-studied for use in gene expression studies [1-3]. Finding an optimal subset of features is combinatorially prohibitive, so RFE reduces the complexity of feature selection by being "greedy". That is, once a feature is selected for removal, it is never reintroduced. Most studies have found RFE to select very good gene sets, but with 12000 or more genes to select from, when the number of samples (patients) is large, RFE takes considerable computation time. Recursive feature elimination is extremely computationally expensive when only *one *least useful feature is removed during each iteration. A modified version of RFE, RFE-Annealing, is proposed here, aimed at greatly reducing the computational time required to perform the RFE ranking process while maintaining comparable performance with respect to prediction accuracy. Instead of removing only one feature at a time, RFE-Annealing removes a set of features each time, with the number of features removed decreasing in each iteration. As its name implies, the process is similar to the well-known method of simulated annealing [4-6].

Simulated annealing has its roots in metallurgy and thermodynamics. Annealing is used in metallurgy to create materials with fewer defects. In thermodynamics, annealing is used to find an optimal state which has minimum energy. The basic process has two steps: heat to a high temperature and then cool very slowly. The annealing schedule, which is the heart of the process, defines how to reduce the temperature during the cooling phase. Simulated annealing is a metaheuristic (not problem specific) that is used in many combinatorial optimization problems. Combinatorial search techniques have difficulty distinguishing between *local *minima (or maxima) and *global *minima (or maxima). Simulated annealing is used in a search by selecting a random state to start and using the annealing schedule to guide the search. Early in the search there is a higher probability of making a move in the search space to a solution that is worse than the one before. This is appropriate since the initial solution was random and the optimal solution may be far off. As the search proceeds, the probability of making a move to a worse solution is decreased slowly. The temperature decrease corresponds to the decreasing probability of moving to a worse solution. RFE-Annealing uses the annealing schedule idea to remove a large number of genes in the initial iterations (when it is easy to identify unimportant genes). In later iterations, the number of genes removed is reduced so that important genes are not removed. The simple schedule of removing  of the remaining genes during iteration *i *is used. That is, half are removed in the first iteration, one-third in the second, one-fourth in the third, and so on. Details of the algorithm can be found in the Methods section.

Vladimir Vapnik invented Support Vector Machines (SVMs) in 1979 [7]. SVMs often achieve superior classification performance compared to other learning algorithms across most domains and tasks. They are efficient enough to handle very large-scale classification in both number of samples and number of variables [8]. SVMs are generated in two steps. First, the data vectors are mapped to a high-dimensional space. Second, the SVM tries to find a hyperplane in this new space with maximum margin separating the classes of data. Sometimes it is not possible to find a separating hyperplane even in a very high-dimensional space. In this case, a trade-off is introduced between the size of the separating margin and penalties for every vector that is within the margin [9]. The margin denotes the distance from the boundary to the closest data point in the feature space.

**Figure 1:**
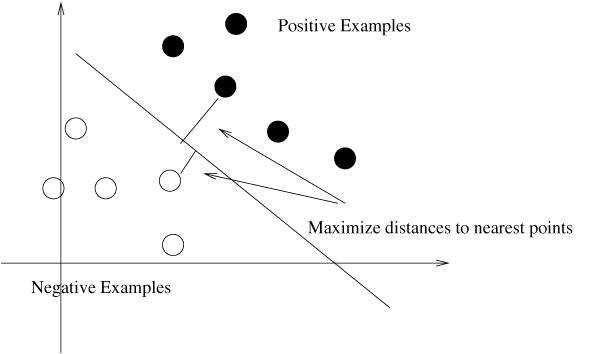
**A Linear Support Vector Machine [16]**. In its simplest, linear form, a SVM is a hyperplane that separates two classes of examples (postive and negative) with maximum margin. The margin is defined by the distance from the hyperplane to the nearest of the data points.

In its simplest, linear form, a SVM is a hyperplane that separates two classes of examples (postive and negative) with maximum margin (see Figure 1). The SVM creates and outputs a weight vector, where each dimension (feature) is assigned a weight. The weight vector is used to determine the least important feature, which is defined to be the one with the *smallest *weight in the weight vector. The least important feature is selected for removal in each iteration of the recursive feature elimination procedure.

## Results and Discussion

### Results

#### Data Sets

We analyzed three well known data sets: 1) the data of Bhattacharjee *et al*. [10], which is a set of 12,600 gene expression measurements (Affymetrix oligonucleotide arrays) per patient from 203 patients with normal subjects and four subtypes of lung carcinomas; 2) a colon cancer data set [11], consisting of expression levels of 2000 genes describing 62 samples (40 tumor and 22 normal colon tissues, Affymetrix oligonucleotide array); 3) a pediatric Acute Lymphoblastic Leukemia (ALL) data set from St. Jude Children's Research Hospital (SJCRH) [12], which includes 12,625 gene expression measurements (Affymetrix arrays) per patient from 246 patients with six different subtypes of ALL. In the research of SJCRH, 246 cases of pediatric ALL were analyzed on the U133 A and B chips. There are six primary subtypes of ALL involved in the study: BCR-ABL, E2A-PBX1, Hyperdiploid>50, MLL, T-ALL and TEL.

#### Model

The three data sets were used to compare the performance of the RFE and RFE-Annealing algorithms. Each data set was randomly divided into stratified training and hidden test sets. A different number of genes was selected by each of the algorithms. The SVM was trained on the training data that was trimmed to the selected genes from each algorithm respectively. The SVM model produced was evaluated by its performance on the hidden test data to predict the class labels (since cross validation results on the training data tend to be optimistic). More details of the model design are given in the Methods section. Comparisons of the two algorithms in terms of prediction rate and time required are made. A comparison between RFE-Annealing and SQRT-RFE [2,3] is also performed. Like RFE-Annealing, the goal of SQRT-RFE is to improve the performance of RFE by removing a set of features during each iteration. In that algorithm,  features are removed at each step.

#### Experimental results on the SJCRH data

The performance of RFE-Annealing was comparable to both the RFE and SQRT-RFE algorithms in terms of prediction accuracy rate (each achieving around 98%-100% accuracy on the test data), but RFE-Annealing is much less computationally intensive than the RFE algorithm. RFE-Annealing took approximately 26 minutes for gene selection, while the RFE algorithm spent around 58 hours, and SQRT-RFE required 1 hour to complete. All the experiments were run separately on an unloaded machine (with 2.6GHz dual processors and 2GB memory).

**Figure 2:**
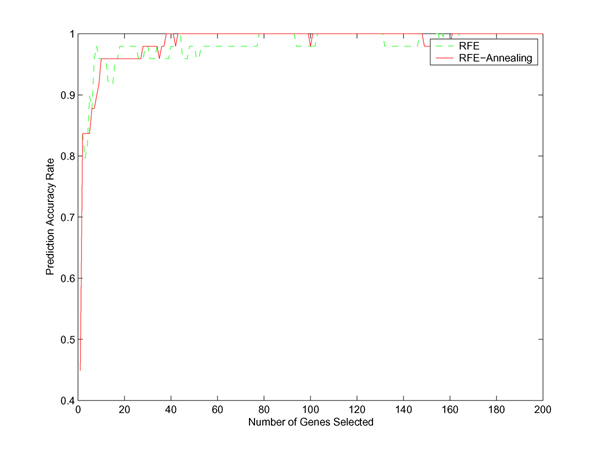
**Comparison of RFE and RFE-Annealing in terms of Prediction Rate on SJCRH data**. This figure shows the results of comparing RFE and RFE-Annealing using the St. Jude Children's Research Hospital ALL study. Accuracy rates on the hidden test set are very high for both gene selection algorithms across all gene set sizes up to 200.

Figure 2 shows a comparison of RFE and RFE-Annealing. We can see that in most of the cases when the number of genes ranges from one to 200, RFE-Annealing performs at least as well as the original RFE. Also it is more stable than the original RFE. Only in a few points (e.g. when the number of genes selected is around 10), does RFE give a slightly higher prediction rate than RFE-Annealing.

**Figure 3:**
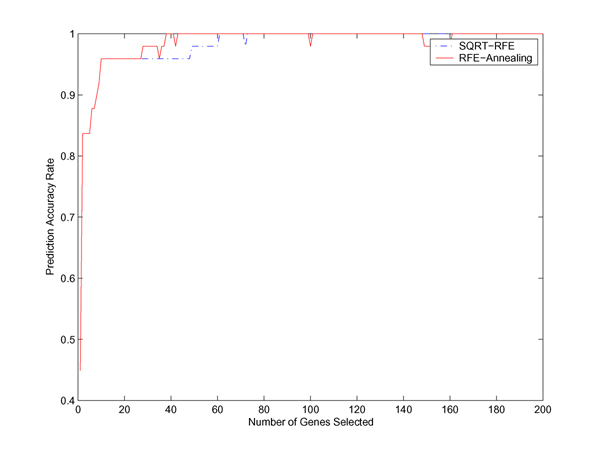
**Comparison of SQRT-RFE and RFE-Annealing in terms of Prediction Rate on SJCRH data**. This figure shows the results of comparing SQRT-RFE and RFE-Annealing. They are very comparable with respect to accuracy on the hidden test data.

When compared to SQRT-RFE, RFE-Annealing has comparable performance in terms of prediction rate. See Figure 3.

#### Experimental results on Bhattacharjee and Alon data

**Figure 4:**
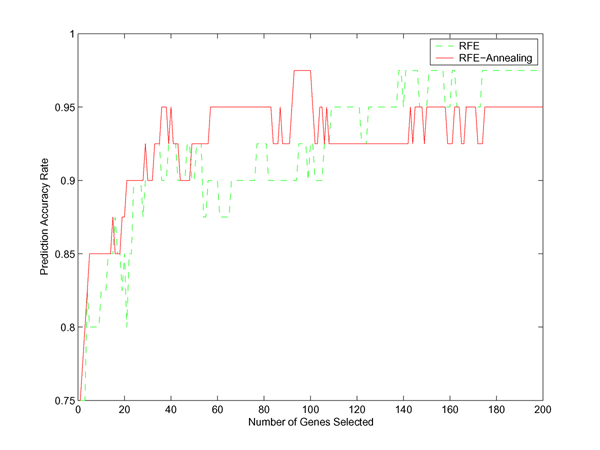
**Comparison of RFE and RFE-Annealing in terms of Prediction Rate based on Bhattacharjee Data**. When comparing RFE and RFE-Annealing on the hidden test data from the Bhattacharjee set, the algorithms both do well. RFE does slightly better when more than 100 genes are selected and RFE-Annealing does slightly better when less than 100 genes are used.

**Figure 5:**
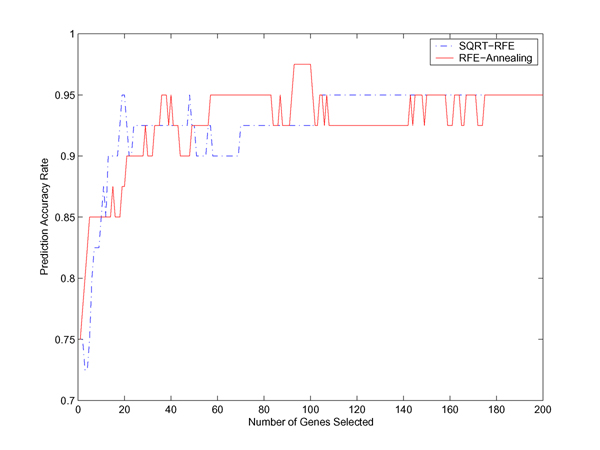
**Comparison of SQRT-RFE and RFE-Annealing in terms of Prediction Rate based on Bhattacharjee Data**. The accuracy rates are very similar when comparing SQRT-RFE and RFE-Annealing on the hidden test data from the Bhattacharjee set.

Experiments on the Bhattacharjee and Alon colon cancer data sets also show that RFE-Annealing has similar performance when compared with both RFE and SQRT-RFE with respect to accuracy. The computational requirement of RFE-Annealing is slightly less than SQRT-RFE, and significantly less than the original RFE. Figures 4 and 5 show that when tested on the Bhattacharjee data, in many cases RFE-Annealing outperforms the other two, but in some cases, it performs slightly worse. RFE-Annealing seems to perform slightly better when fewer than 100 genes are selected and RFE performs slightly better when 100 or more genes are selected.

**Figure 6:**
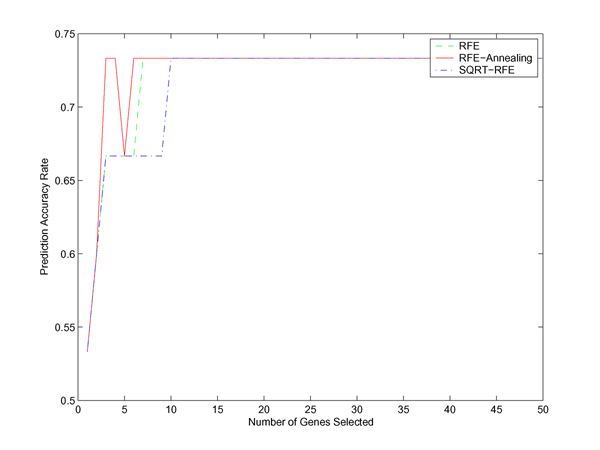
**Comparison of RFE, RFE-Annealing and SQRT-RFE in terms of Prediction Rate based on Alon's Data**. On the smaller Alon data set, all three gene selection methods yielded the same accuracy on the hidden test data when 10 or more genes were selected. The largest gene set selected was 50 due to the fewer number of genes and samples.

When tested on Alon's colon cancer data, Figure 6 shows little difference between RFE-Annealing and the other two algorithms in terms of prediction rate performance. But when comparing computational time, RFE-Annealing is much faster than the other two. Results are shown in Table 1. Even with a small data set like Alon, with 62 samples of around 2000 genes, there is a significant difference between the time required by RFE-Annealing and RFE, at 30 seconds and 6 minutes 18 seconds, respectively. SQRT-RFE required one minute on the same data set. For the larger data sets the difference is even more dramatic. In general, the three algorithms selected very similar "best" gene subsets, which also shows that these three algorithms are comparable. In the SJCRH data, 187 out of 200 genes are in common between RFE and RFE-Annealing algorithms, while 189 between RFE-Annealing and SQRT-RFE.

In the Bhattacharjee data, 182 out of 200 genes are the same from the RFE and RFE-Annealing algorithms. Similarly, 185 are the same for RFE-Annealing and SQRT-RFE.

In the Alon data, there are 47 out of 50 genes in common between RFE and RFE-Annealing and 46 out of 50 between RFE-Annealing and SQRT-RFE.

### Biological analysis

In order to investigate the biological meaning of the selected important genes in the SJCRH data, table 2 shows the pathways derived from NetAffx [13] to which the 200 genes selected by the algorithms belong. RFE and RFE-Annealing are very similar in the pathways represented by the genes selected. The primary differences are between SQRT-RFE and the other two algorithms. The pathways "Calcium regulation in cardiac cells", "G Protein Signaling", "Inflammatory Response Pathway", "Inositol Phosphate metabolism", and "Smooth muscle contraction" have the least consistency.

## Conclusion

In general, RFE-Annealing allows an enormous increase in the efficiency of the algorithm without a decrease of classification accuracy. Thus the gene selection process is made much more practical in domains with a large number of features, such as gene expression data. This improvement is especially important as the number of samples available increases.

## Methods

### SVM-RFE-Annealing

Guyon introduced Recursive Feature Elimination (RFE) for Support vector machines (SVM) in [1]. SVM-RFE performs feature selection by iteratively training a SVM classifier with the current set of features and removing the least important feature indicated by the SVM [14]. In the linear case, the separating hyperplane (decision function) is *D *() = () + *b*. The feature with the smallest weight *w*^2 ^contributes the least to the resulting hyperplane and can be discarded. Due to the heavy computational cost of RFE, several variants have been introduced to speed up the algorithm. Instead of removing only one least important feature at every iteration, removing a big chunk of features in each iteration will speed up the process. The goal is to remove more features during each iteration, but not to eliminate the important features. SQRT-RFE removes  features at each step, where *S *is the number of remaining features in each iteration. Another variant is Entropy-based RFE (E-RFE) which eliminates features based on the structure of the weight distribution [2,3]. An entropy function *H *is introduced as a measure of the weight distribution.

We introduced RFE-Annealing which eliminates larger sets of features at the beginning steps, but as the algorithm proceeds, the number of features removed is reduced in each step. The basic idea is: in the *n*th iteration, train a SVM on the active features and remove l/(*n *+ 1) of least important features. This process continues until only *m *features are left, where *m *is a user defined variable. This method is simple and easy to implement.

**Algorithm SVM-RFE-Annealing: **(Variation of SVM-RFE [1])

Inputs:

Training examples

*X*_0 _= [x_1_, x_2_, ...x_*k*_, ...x_*l*_]*T*

Class labels

**y **= [*y*_1_, *y*_2_, ....*y*_*k*_, ....*y*_*l*_]*T*

Initialize:

Subset of surviving features

**s **= [l, 2, ...n]

Feature ranked list

**r **= []

Repeat until **s **= []

Restrict training examples to good feature indices

*X *= *X*_0_(:, **s**)

Train the classifier

α = *SV M *- *train*(*X*, **y**)

Compute the weight vector of dimension length(s)

**w **= Σ_*k*_α_*k*_*y*_*k*_**X**_*k*_

Compute the ranking criteria

*c*_*i *_= (*w*_*i*_)^2^, for all i

Repeat *length*(*s*) ×  times

Find the feature with smallest ranking criterion

*f *= *argmin*(**c**)

Update feature ranked list

**r **= [**S**(*f*), **r**]

Eliminate the feature with smallest ranking criterion

**s **= **s**(l : *f *- 1, *f *+ 1 : *length*(**s**))

end

end

Output:

Feature ranked list **r**.

### Models

**Figure 7:**
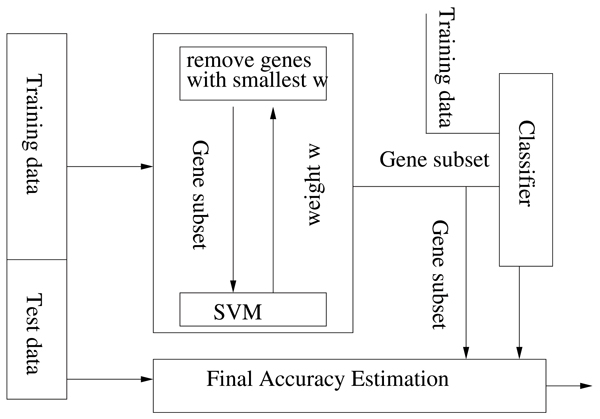
**Model Design**. This figure describes the methodology used in the testing. The methodology employs a similar wrapper technique as described in [17]. The data sets were first divided, in a stratified way, into training and hidden test data (80% training and 20% test, except for Alon which was 75%/25% since it was smaller). The weight vector from the SVM with a linear kernel was used to identify the gene(s) to remove. The training data was projected down to just the specified subset and a classifier was constructed using a linear SVM. Then the test data was projected to the same feature set and tested using the classifier built from the training data. This was repeated for each data set, for each algorithm and for each size feature set from 1 to the maximum features selected.

Data is split into training data and test data with proportion 80% training and 20% test (except the smaller Alon data, which is split with 75%/25%). As shown in Figure 7, based on training data, a linear SVM is built. The implementation of SVM in Weka [15] was used for all testing. Genes with the smallest weight are removed iteratively, according to the gene selection algorithms (RFE, RFE-Annealing and SQRT-RFE). We will have *m *genes left, where *m *is a user defined parameter, and the ranking of genes is based on the order they are removed. If *m *= 0, we will get the ranking of all of the genes. The user can select the *m *most important genes and build a SVM classifier based on the training data. The SVM is then run on the test data and an estimated prediction accuracy rate is obtained. Since running the RFE algorithm on large data sets like the SJCRH data takes more than 50 hours, this train/test process was performed only once on each data set. The algorithms are deterministic, so the accuracy is fixed for the particular train/test split used. The machine was unloaded except for the particular program being tested, so the time estimates are relatively stable.

In our experiments, we let *m *= 1, 2, ...200 for the SJCRH and Bhattacharjee data sets and *m *= 1, 2, ....50 for the Alon data, since the Alon data only had 2000 genes initially and the other two had more than 12,000 genes.

In order to test the performance of the RFE-Annealing algorithm, for each *m, m *genes were selected by RFE, SQRT-RFE and RFE-Annealing respectively, and the SVM models were built and the prediction rates on the test data were compared. The overall process is explained in the diagram in Figure 7.

## Authors' contributions

YD contributed to writing the computer code. DW guided the analysis. Both authors contributed to the development of methodology and writing the manuscript. Both authors read and approved the final manuscript.

## Figures and Tables

**Table 1:** Comparison of RFE, RFE-Annealing and SQRT-RFE algorithms in terms of time

Data	Number of samples	RFE	RFE-Annealing	SQRT-RFE
St.Jude	246	58 hours	26 minutes	60 minutes
Bhattacharjee	203	27 hours	20 minutes	38 minutes
Alon	62	6.3 minutes	0.5 minute	1 minute

This table clearly demonstrates the computational efficiency of RFE-Annealing over SQRT-RFE and especially RFE. When data sets with a large number of samples and a large number of genes (e.g. SJCRH and Bhattacharjee) are used the difference is substantial.

**Table 2:** Pathways associated with the selected genes in SJCRH data

Pathway	RFE	RFE- Annealing	SQRT-RFE
Apoptosis	2	2	2
Apoptosis_GenMAPP	3	3	3
Apoptosis_KEGG	2	2	2
Arginine and proline metabolism	2	2	2
Biosynthesis of steroids	1	1	1
Calcium signaling pathway	19	18	17
**Calcium_regulation_in_cardiac_cells**	6	4	4
Cell_cycle_KEGG	8	8	9
Cholesterol_Biosynthesis	1	1	1
Circadian_Exercise	2	2	2
DNA_replication_Reactome	0	0	1
Electron_Transport_Chain	1	1	1
Fructose and mannose metabolism	1	1	1
Gl_to_S_cell_cycle_Reactome	6	6	7
**G_Protein_Signaling**	6	4	4
Galactose metabolism	1	1	1
Glutathione metabolism	1	1	1
Glycerolipid metabolism	1	1	1
Glycerophospholipid metabolism	3	3	3
Glycine, serine and threonine metabolism	2	2	2
Glycolysis/Gluconeogenesis	1	1	1
Glycolysis_and_Gluconeogenesis	1	1	1
GPCRDB_Class_A_Rhodopsin-like	0	1	0
Hypertrophy _model	1	1	1
**Inflammatory_Response_Pathway**	1	1	4
**Inositol phosphate metabolism**	4	4	0
Integrin-mediated_cell_adhesion_KEGG	4	4	4
MAPK_Cascade	1	1	1
mRNA_processing_Reactome	2	2	3
Nicotinate and nicotinamide metabolism	1	1	1
Ovarian_Infertility_Genes	3	3	3
Oxidative phosphorylation	1	1	1
Pentose phosphate pathway	1	1	1
Phosphatidylinositol signaling system	4	4	3
Prostaglandin_synthesis_regulation	1	0	1
Proteasome_Degradation	1	1	1
Purine metabolism	3	2	2
Pyrimidine metabolism	2	2	2
**Smooth_muscle_contraction**	12	10	10
Statin_Pathway_PharmGKB	0	0	1
TGF_Beta_Signaling_Pathway	4	4	4
Terpenoid biosynthesis	1	1	1
Type I diabetes mellitus	4	5	5
Ubiquitin mediated proteolysis	2	2	2
Urea cycle and metabolism of amino groups	2	2	2
Wnt_signaling	3	3	2

This table lists the various known pathway associations with the size 200 gene sets selected by the algorithms using the SJCRH data. The values in the table represent the number of genes associated with that pathway in each of the sets. In general there was considerable overlap in the genes selected (over 90%). The pathways with the least consistency are shown in boldface. Pathway data was derived using NetAffx [13].
